# Parental intervention strategies and operating mechanism on adolescent social media use—The concept of literacy improvement based on interaction

**DOI:** 10.3389/fpsyg.2022.1043850

**Published:** 2022-12-01

**Authors:** Bowei Wang, Jiali Chen

**Affiliations:** School of Journalism and Communication, Nanchang University, Nanchang, China

**Keywords:** usage behavior knowledge, literacy improvement, social media, parent-mediated intervention, online friends

## Abstract

This study focuses on a realistic picture of parental intervention in the use of social media among teenagers in the post-pandemic era. First, based on a questionnaire survey and in-depth interviews, and under the guidance of the concept of interactive literacy improvement, we propose a conceptual model and a verifiable measurement dimension of parental-mediated intervention. Second, based on the comparison of parent–child samples, it was found that parental-mediated intervention strategies are often used in families, and parents and children have roughly the same cognition and preference for the four intervention strategies. However, parents reported that they use intervention strategies much more frequently than perceived by their children. Third, we constructed and verified the prediction model of “individual technical characteristics-online family environment-parental-mediated intervention,” namely, the hierarchical progressive logic of parental-mediated intervention, and realized the systematization of influencing factors. The study confirmed that the effectiveness of parental-mediated intervention is improved by parent–children “cohesion.” In the follow-up, we will focus on the new perspective of theoretical research on parental intervention and intra-generational digital inequality among adolescents.

## Introduction

The Covid-19-induced need for “social distancing” and emergency adoption of “online courses” has contributed to people’s, especially teenagers’, obsession with online social interaction. Forty percent of teenagers (CNNIC, 2020) have either been exposed to undesirable online content, such as pornography, violence, and bloodshed, or have experienced psychological and behavioral disorders, such as cyber bullying, cyber violence, irrational consumption, privacy disclosure, and addiction to online games and short videos. The newly revised 2021 law for the protection of minors in the People’s Republic of China has added a chapter on “network protection,” requiring Internet platforms to launch or upgrade the “youth model.” “Youth model” is a system that regulates adolescent online behavior in terms of use period, duration, functions and browsing content, which applied to short video platform, social platform, game platform and other network platforms. However, technological settings alone cannot ensure that the youth use the Internet in a civilized, productive, and healthy manner.

Parents are the “first line of defence” for children against undesirable content (Liu et al., 2022a). The guidance, restriction, and regulation of children’s media use behavior constitutes their digital parenting responsibilities and obligations (Jones et al., 2013). With the progress of communication technology and changes in the media environment, teenagers have identified ways to evade parental monitoring (Liu et al., 2022b), such as erasing browsing history, deleting instant messages, and installing location masking software. To deal with a series of new situations and problems emerging in this context, this study focuses on the realistic picture of parental intervention in the post-pandemic era by adopting the individual–environment–behavior theory as the analytical framework. We innovatively consider the technological characteristics of parents and the online family environment jointly constructed by parents and children as antecedents to explain parental intervention behavior from both logical and empirical perspectives. We also clarify the mechanisms of parental intervention.

## Theoretical background and hypothesis building

### Guiding philosophy and strategies of parental intervention

It is not clear whether the influence of media on teenagers is largely positive or negative. The final influence, it seems, depends on how parents intervene and guide their children’s media use behavior (Clark, 2011). According to Desmond et al. (2010), who first defined this phenomenon as “parental intervention” in 1985, parents explain the complexity of the social environment presented by television media in a manner that is intelligible to their children, so as to promote their cognitive development. Chinese scholars have generally recognized the important role of parents in shaping the media use behavior of their children as can be seen in related research on topics, such as “parent intervention” (Chen, 2019), “parent control” (Liu, 2017), “parental intervention” to protect their children from undesirable media content (Zeng and Zhan, 2022; Zheng, 2022); “new media education for parents” focusing on media education and education action (Liu and Huang, 2018). Research has also been done on the premise that parents know about the media use behavior of their offspring (Ding et al., 2019) on topics such as “parental monitoring” and “parental network supervision” (Su et al., 2016; Zhang et al., 2020).

A review of studies on the Internet, social media, online games, and other interactive technologies shows that research on parental intervention is rooted in the research tradition of the media effect theory. Accordingly, parental intervention is interpreted as parents’ conscious management and regulation of media and content accessible to their children (Lee and Chae, 2012), to regulate and weaken the adverse effects of media use on teenagers (Shin and Huh, 2011). Thus, the guiding concept of parental intervention is the protection of children from “undesirable influence,” with parents playing the role of protector (Waizenhofer et al., 2004).

Parents are the gatekeepers for their children’s media use. However, given the popularity of mobile Internet, widespread use of digital devices, and loss of adults’ absolute control over information, an increasing number of families are exhibiting a trend of “de-autocracy” in children’s media use. However, research on parent-mediated intervention still adheres to the guiding concept of ensuring children’s “safety of use,” which needs to be urgently developed into the concept of interactive literacy improvement. Therefore, in this study, parent-mediated intervention—in which parents integrate into children’s online activities as co-participants—is a dual interaction of emotion and behavior aimed at improving children’s digital literacy. Positive, restricted, and joint viewings were the three dominant strategies of parent-mediated intervention in the era of TV media (Austin, 1993); they are still widely adopted in the follow-up intervention of interactive technologies, such as in online games—albeit less effectively than television intervention. Additionally, the cognition and favor of parent–child samples on parent-mediated intervention strategies in the use of TV media are roughly the same (Nathanson, 2001). Consequently, we raised our first research question.

*RQ1*: In the post-epidemic era, how do parents interfere with children’s social media use and what strategies do they use? What are the cognitions and preferences of parents and children regarding different intervention strategies?

### Predictive model of parental-mediated intervention strategies

Kurt Lewin, a German psychologist, proposed that predicting individual behavior requires attention to the individual, the environment, and the interaction between the two. This viewpoint provides a theoretical framework for predicting parents’ intervention in children’s social media use. Additionally, established studies on the factors influencing parental intervention provide explanatory variables. How parents intervene in their children’s media use is rooted in their attitudes toward media (Warren, 2001). Established research confirms that when parents focus more on the negative effects of television or games, they implement a mix of intervention strategies (Shin and Huh, 2011), especially when they compulsively restrict their children’s use behavior (Carl et al., 1981). Parents are more inclined to use co-viewing or co-using interventions when they believe that watching or using media will expand their children’s knowledge and enhance their cognitive abilities (Nathanson, 2001). Accordingly, we propose the following hypothesis:

*H1*: Parental attitudes toward social media can predict their interference with children’s social media use.

Parents’ ability to use media is a prerequisite for effective performance of parental intervention duties (Austin, 1993); parents with poor media use skills are more likely to employ permissive or coercive interventions, such as outright confiscation of cell phones and disconnection from the Internet (Lee, 2013). The frequency of parents’ Internet use also significantly predicts their choice of intervention (Livingstone and Helsper, 2007). Parents who are obsessive or active users of television or online games are more likely to formulate rules to restrict their children’s media use in a targeted manner, because they are better informed (Padilla-walker et al., 2011; Liu et al., 2022c). Accordingly, we propose the following hypotheses:

*H2–H3*: Parental proficiency in skills (H2) and frequency of online activity participation (H3) can predict their intervention in children’s social media use.

It is noteworthy that individuals’ attitude toward media impact their usage behavior. Individuals with more positive attitudes tend to participate in a wider range of online activities. If individuals have more positive attitudes, their online activities tend to be extensive (Perazzo et al., 2020). For example, older people who hold a negative perception of online financial applications, such as online banking, will tend to refuse to use them; this will inevitably affect their corresponding usage skills. Accordingly, we propose the following hypotheses:

*H4–H5*: Parents’ attitudes toward social media influence their frequency of online activity participation (H4) and proficiency in skills (H5).

Previous studies have identified that the extent of participation in online activities is not only closely related to age, gender, socioeconomic status, etc. but also the skills used (Liu et al., 2021; Livingstone and Helsper, 2007). Additionally, the richer the online activities performed by an individual, the relatively higher the corresponding level of online skills (Livingstone and Helsper, 2008; Lin et al., 2020; Chen et al., 2021). Accordingly, we propose the following hypothesis:

*H6*: There is a correlation between proficiency in skills and the frequency of online activity participation.

Unlike the traditional media era that relied on the technological environment, the social media era relies on the online family environment jointly constructed by parents and children. The environmental factors influencing parent-mediated interventions were assessed based on this online family environment. In this study, “whether parents and children are online friends” and “parental knowledge of adolescent usage behavior” constituted the bases for assessing the harmony of online family environment. The fact that parent–child subjects are online mutual friends indicates that children are willing to disclose their online lives to their parents to a certain extent (Padilla-walker et al., 2011). Simultaneously, parents’ gradual involvement and integration into their children’s Internet life can enhance their level of knowledge about their children’s online behavior (Wang et al., 2005). In turn, parental information is embedded in parent–child communication, and a few studies have considered parental information as a broad dimension of parent–child relationship quality (Anderson and Branstetter, 2012). Thus, we hypothesized that parents being online friends with their children and their level of knowledge of adolescent usage behavior may influence their preferences for different intervention strategies. Accordingly, we propose the following hypotheses:

*H7*: There is a correlation between parent-child subjects being mutual online friends on social media platforms and parental knowledge of adolescent usage behavior.

*H8, H9*: Parents being their children’s social media friends (H8) and their level of knowledge about their children’s social media use behavior (H9) influence their intervention in their children’s social media use.

In our previous interviews, when asked whether their parents were aware of their social media use, 82.2% of respondents said “yes,” 5.8% said “no,” and 12% were “not sure.” The comparison suggested that parents who were better informed about their children’s online behavior tended to have a more positive attitude toward social media and a relatively practiced skill set. When asked whether their parents were their social media friends, 30% of children responded that their parents were their online friends on all social apps; such parents participate in a relatively wide range of online activities. A total of 38.7% said that their parents were their online friends on a few social platforms, partly because they did not use them and partly because they deliberately hid them from their parents. As can be seen, the interview data partially confirm a correlation between parents’ technological characteristics—attitudes toward social media, frequency of online activity participation, and proficiency in skills—and the construction of an online family environment—online mutual friends, informed use behaviors, etc. However, inadequacy of empirical data is a major challenge for the current study. Accordingly, we propose the following hypotheses:

*H10–H12*: Parents’ attitudes toward social media (H10), frequency of online activity participation (H11), and proficiency in skills (H12) influence parents’ intervention in children’s social media use by facilitating online mutual friendship.

*H13–H15*: Parents’ attitudes toward social media (H13), frequency of online activity participation (H14), and proficiency in skills (H15), in turn, influence their intervention in children’s social media use by promoting parental awareness about children’s media use behaviors.

**Figure 1:**
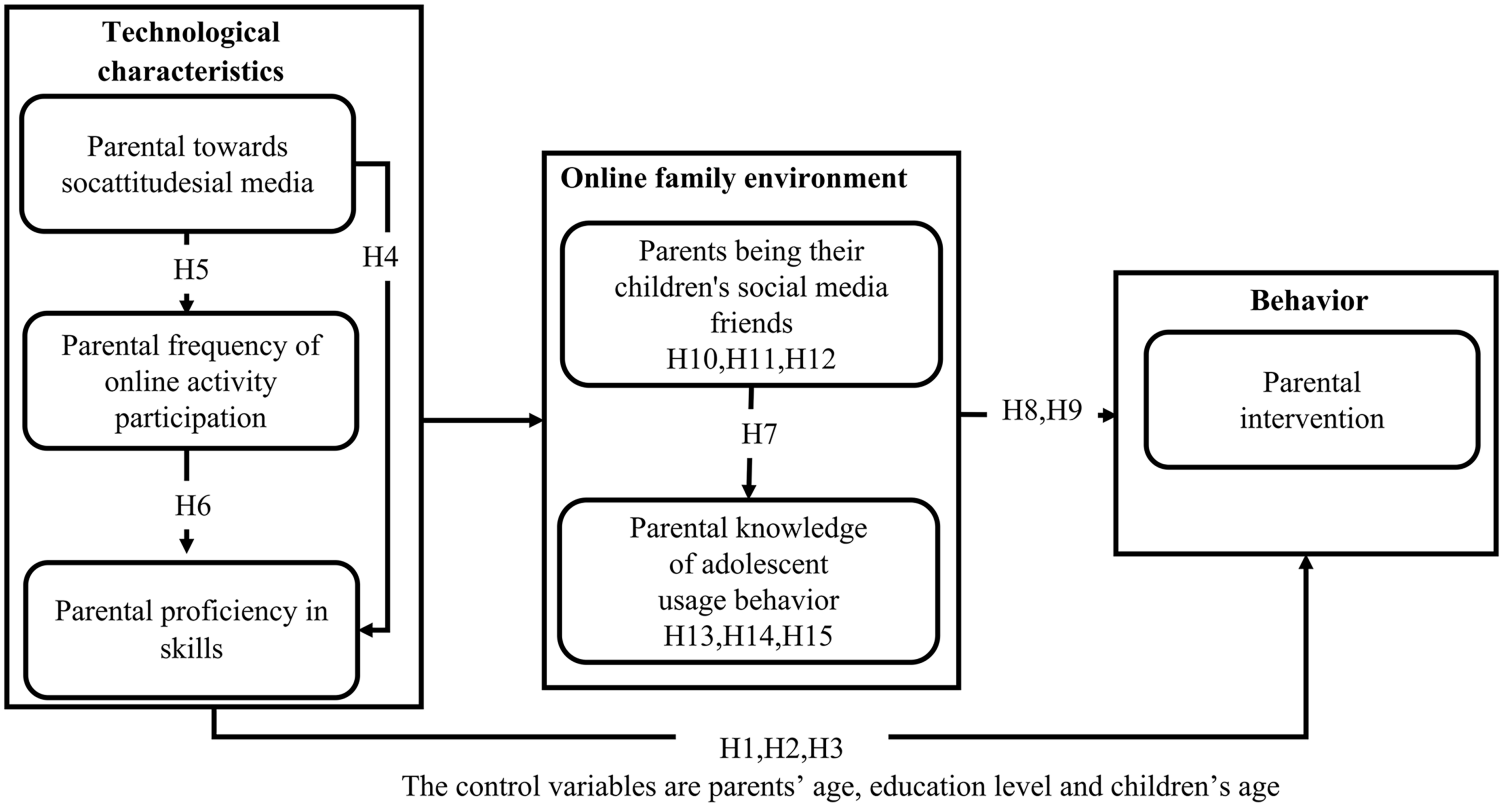
Predictive model of parental intervention Strategies.

Based on the 15 research hypotheses mentioned above, an attribution model was proposed to explain the adoption of parental intervention strategies (Figure 1).

## Methodology

### Data collection and analysis

Our study examined parents’ intervention in adolescents’ social media use in the post-pandemic era and explored the mechanisms influencing their adoption of specific intervention strategies. Middle school, high school, junior college, and technical school students aged 12–17 were specified as youth. According to Van den Bulck and Van den Bergh (2000), social responsibility norms induce parents to intentionally exaggerate their media intervention in questionnaires. In contrast, children may intentionally weaken their parents’ intervention for peer admiration (Warren, 2001). Consequently, this study incorporated semi-structured online interviews with 40 parent–child paired samples, in addition to the parent sample questionnaire, to present a clear and complete picture of parental interventions from the dual perspective of parent–child subjects.

The data collection of the parental sample was selected from Nanchang, Shangrao and Ganzhou, Jiangxi Province, in Mainland China. Using PPS sampling, 24 sampling units were obtained based on the principle of equal spacing by ranking economic indicators, and one community was selected from each sampling unit, and 20–25 households were randomly selected in each community. Parents were surveyed if there were adolescents aged 12–17 in the sampled households; if not, scroll down and try to ensure a 50/50 split of male and female respondents. The survey was conducted in face-to-face interviews between December 1 and 30, 2021, the newly revised 2021 law has just been issued. The questionnaires were manually reviewed after collection. The questionnaires with too many “not sure” and “cannot say” options were eliminated.500 questionnaires were distributed, 469 were collected, 447 were valid, and a valid renturn rate was 89.40%.

In terms of parental sample composition, 47.1 and 52.9% of the respondents were male and female, respectively; 44.7% were aged 41–45, 36.3% were aged 36–40, and the remaining three age-groups accounted for 19.0% in total. A total of 21.7% were in junior high school and below, 26.2% were in technical secondary school / senior school / technical school, 28.9% were in college, 16.2% were university undergraduates, and 7% were postgraduates and above. The participants included professional and technical personnel (28.9%), employees of enterprises and companies (25.4%), staff of party and government organs (18.2%), freelancers (14.0%), and agricultural, forestry, and fishery production workers (13.5%). A total of 54.8% of the participants self-assessed their family economic level as middle, 21.5% as middle and low, 14.7% as middle and high, while 9% were not sure about their family income level. If there are several children fit the age range, the first child was selected to fill in the questionnaire. The children of the respondents were as follows: 33.5% were 12–13 years old, 35.6% were 14–15 years old, and 30.9% were 16–17 years old.

### Measurements

#### Parents’ attitudes toward social media

Referring to the scale of Yi and Lin (2015), five items were measured; for example, “Social media is very interesting,” “My work and life would be very different without social media,” etc. Responses ranged from “strongly disagree” = 1 to “completely agree” = 5 (M = 2.43, SD = 1.04, α = 0.90).

#### Frequency of online activity participation

The use of eight service functions was measured; for example, “search (people, groups, official accounts),” “give a like, comment, dynamic publishing,” etc. (Wei et al., 2011; Zhou, 2014; Liu et al., 2022d). The responses ranged from “not at all” = 1 to “often” = 5 (M = 3.35, SD = 1.03, α = 0.89).

#### Proficiency in skills

Referring to Jianhua, van Deursen, and van Dijk’s scale, we focused on users’ mastery of six skills, such as “creating content” and “security settings;” responses ranged from “unfamiliar” = 1 to “very familiar” = 5 (M = 3.76, SD = 1.35, α = 0.87; van Deursen and van Dijk, 2010).

#### Parents being online friends of their children

The responses of the proactive invitee and the invitee were examined with the help of three questions based on the study by Katherine and Joy (2011), such as “How did you react when your child invited you to become an online friend?”; responses were scored on a 5-point Likert scale, ranging from “very unhappy” = 1 to “very happy” = 5 (M = 3.18, SD = 1.14, α = 0.94).

#### Parental knowledge of adolescent usage behavior

Referring to the Parental Information Scale (Spoth et al., 1998), five items, including “which social media the child uses” and “with whom the child communicates online” were examined; scores ranged from “not at all” = 1 to “very clear” = 5 (M = 2.89, SD = 0.89, α = 0.83).

Cronbach’s alpha values of the above variables were in the range of 0.87–0.94, the KMO of all variables was >0. 83, Bartlett’s sphericity test reached a significant level, and the factor loadings of all items on the corresponding latent variables were greater than 0.50. This indicates that the scale has good structural and functional validities.

#### Parental intervention strategies

A pool of entries for the parental intervention scale was developed after preliminary in-depth interviews and an open-ended questionnaire survey. After two rounds of expert consultation, a 40-item parental intervention scale was finalized, including “Talking to your child about your experiences with using” and “Talking to your child about how to deal with online harassment.” In the survey, parents were asked to respond using a 5-point Likert scale, where 1–5 represented “almost never,” “once or twice a month,” “once or twice a week,” “three or five times a week,” and “almost every day,” respectively (M = 3.34, SD = 0.89, α = 0.87).

After three rounds of exploratory factor analysis of the sample data—and after removing question items 13, 39, and 40—four common factors were extracted. The factor loading values of similar topics constituting each factor were greater than 0.5 (0.54–0.98) and cumulatively explained 69.29% of the variance in the parental intervention scale. Validated factor analysis showed that all 4 s-order factors fit the data well, and the exogenous observed variables fell within the corresponding four common factors, indicating good convergent validity for all four dimensions of parental intervention. By summarizing the commonalities of the 4 s-order factors, conceptual dimensions of rule-based technical enhancement, active guidance enhancement, informed participation enhancement, and two-way empowerment enhancement were proposed. Rule-based technical enhancement means that the parent and offspring jointly develop rules or the parent control, manage and regulate of the social media use behavior of the offspring by technology. Active guidance enhancement refers to promote effective use of social media through guidelines such as “how to distinguish between virtual and real” or “how to achieve safe sharing of content.” Informed participation enhancement refers to achieve parental knowledge of the offspring’s online behavior through integration with the offspring in the online world of social media, then enhancing digital literacy. Two-way empowerment enhancement can be understood as the parent and child encouraging each other to explore and learn new things together on social media, forming family discussion groups to accomplish specific tasks together, sharing problems and interesting things, exchanging insights and judgments about social media use in collaboration. Based on related theories and the dimensional structure of existing measurement scales, the structural dimensions of the three hypothetical unidimensional, three-dimensional, and four-dimensional models were compared. This comparison confirmed that the four-dimensional model had a better fit for the main indicators (X^2^ = 391.475, df = 241, X^2^/df = 1.625; GFI = 0.972, AGFI = 0.941; RMSEA = 0.041; NFI = 0.913, CFI = 0.923, IFI = 0.976).

#### Control variables

Studies related to television, the Internet, and game interventions have found that gender has less influence on the adoption of parental interventions compared to the age of the child (Livingstone and Helsper, 2008). Additionally, parents who are younger and have a relatively high level of education are more likely to adopt relatively positive interventions such as shared use with their children (Roe, 2000). Thus, the age of parents and children, as well as the education level of parents, were used as control variables.

## Result

### Parent vs. offspring: Recognition and preferences on parental intervention strategies

The in-depth interviews revealed that the younger their children are, the more likely are the parents to choose two intervention strategies: actively guided promotion and rule-based technical promotion, to prevent young children from becoming addicted to social media. Parents will give younger children specific instructions on “what” to use and “how” to use social media, discuss and enforce rules with elder children together, or agree to use technologies such as electronic nannies and teenage models to restrict the use of specific functions and permissions. “I limit the amount of time my younger son(age 12) uses social media. But to his elder brother (age 16), I prefer to advice rather than restrict (PR1, male, age 37, master’s degree, interview log P21).”

Parents will also adjust specific intervention methods according to their children’s characteristics; for example, to weaken the recalcitrance of adolescence, they often send motivational “tweets” to guide their children to moderate their use of media(PC1, male, age 37, junior college, interview log P13). For younger children, they arrange diverse family activities to divert their attention(PW2, female, age 38, bachelor’s degree, interview log P74). A few parents also mentioned setting a good example for their children by themselves using the Internet in a reasonable manner(PD3, male, age 44, master’s degree, interview log P105). Most respondents indicated that children’s time limits were the most popular rule-based technology use restriction strategy. Children also reported that “My parents allow me to use social media only on weekends and holidays” (CD1, male, age 13, middle school, interview log P28).

The two-way empowering media intervention strategies require a certain threshold. When parent–child subjects have comparable skills and interact well, and the concept of “members are resources” takes the lead, the “conscious-active-self-sufficient-shared” activity mechanism is followed. With resource sharing and co-construction of active mutual assistance among subjects, instead of one-way passive “metaphor” and “acceptance,” the enthusiasm of parent–child subjects is fully mobilized to improve their digital literacy. “Since I worked with my child on a WeChat car rental, my child(age 16) has stopped calling me ‘technologically illiterate’ and discusses with me all that is new online (PL1, female, age 40, bachelor’s degree, interview log P54).” However, when parents do not have the ability to try new things together with their children, and do not have an inclusive and open mind toward new things, the two-way empowering parental intervention will be hindered.“Mom’s skills are so poor that she is not at the same level as me. Just me teaching her is not a two-way improvement” (CZ2, male, age 12, junior high school, interview log P12).

Informed engagement enhancement strategies are more often a “deviant intervention” after being “selectively informed.” A few parents use online media to stay informed. Parents use Weibo, or re-use QQ, to hope to participate in the daily online life of their teenagers through online parent–children interaction to improve communication and understanding between them. “My mother knows my WeChat and QQ accounts. She added me as a friend and checks my status” (CD3, male, age 17, high school, interview log P104). A total of 73% interviewed parents mentioned that being informed about their children’s online behavior alleviated their anxiety about their children’s online safety and facilitated targeted parental interventions. A total of 53% of the parents felt fully informed about their children’s only use behavior, but 69% of them were only “selectively informed” by their children. “Now I do not talk much on Moments because my mother added me as (her) friend. I prefer to update my status on Weibo or elsewhere” (CW2, female, age 14, junior high school, interview log P78).

Interview data showed that parent–child subjects were relatively consistent in their preference for rule-based technical enhancement, active guidance enhancement, and two-way empowerment enhancement. A few children disagreed with the informed participation enhancement strategy, although most confronted it silently without their parents’ knowledge. For example, “Mom installed location and blocking software on my phone, saying it was for my security. I cracked it off a long time ago, but mom does not know” (CZ2, male, age 17, high school, interview log P12). Parents consider access to their children’s private information with permission as a gatekeeping behavior for online safety, but a few adolescents view it as invasion of privacy. The privacy management theory suggests that adolescents have the right to manage and control access to personal information; however, there is no consensus between parents and children regarding the definition of privacy. In particular, parents do not appropriately relinquish control over their children’s private information as they enter adolescence, as expected by the children (Smetana et al., 2006).

In general, the “four modes” of parental intervention are not singularly presented, but are more often intertwined. A comparison of parent–children samples revealed that parent-children subjects’ perceptions and preferences regarding the types of parental interventions were largely consistent. However, parents reported using their intervention strategies much more frequently than perceived by their children. Thus, our first research question was answered.

### Structural model: Empirical analysis of a predictive model of parental intervention strategies

**Figure 2:**
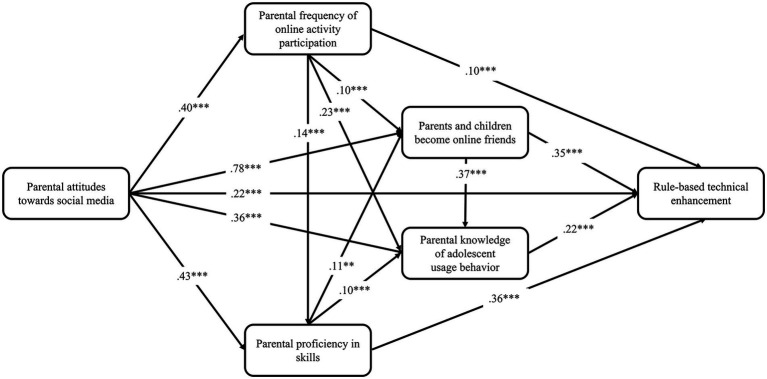
Predictive path coefficients of rule-based technical enhancement. *p < 0.05, **p < 0.01, ***p < 0.001.

**Figure 3:**
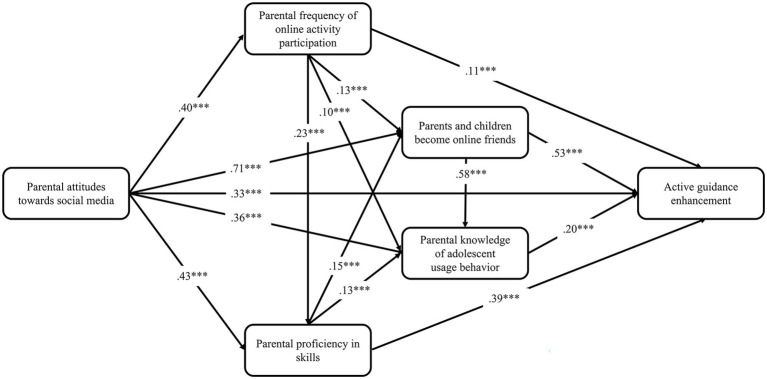
Predictive path coefficients of active guidance enhancement. *p < 0.05, **p < 0.01, ***p < 0.001.

**Figure 4:**
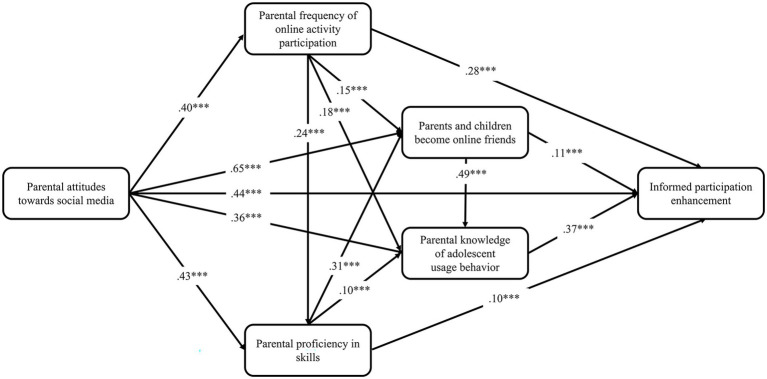
Predictive path coefficients of informed participation enhancement. *p < 0.05, **p < 0.01, ***p < 0.001.

**Figure 5:**
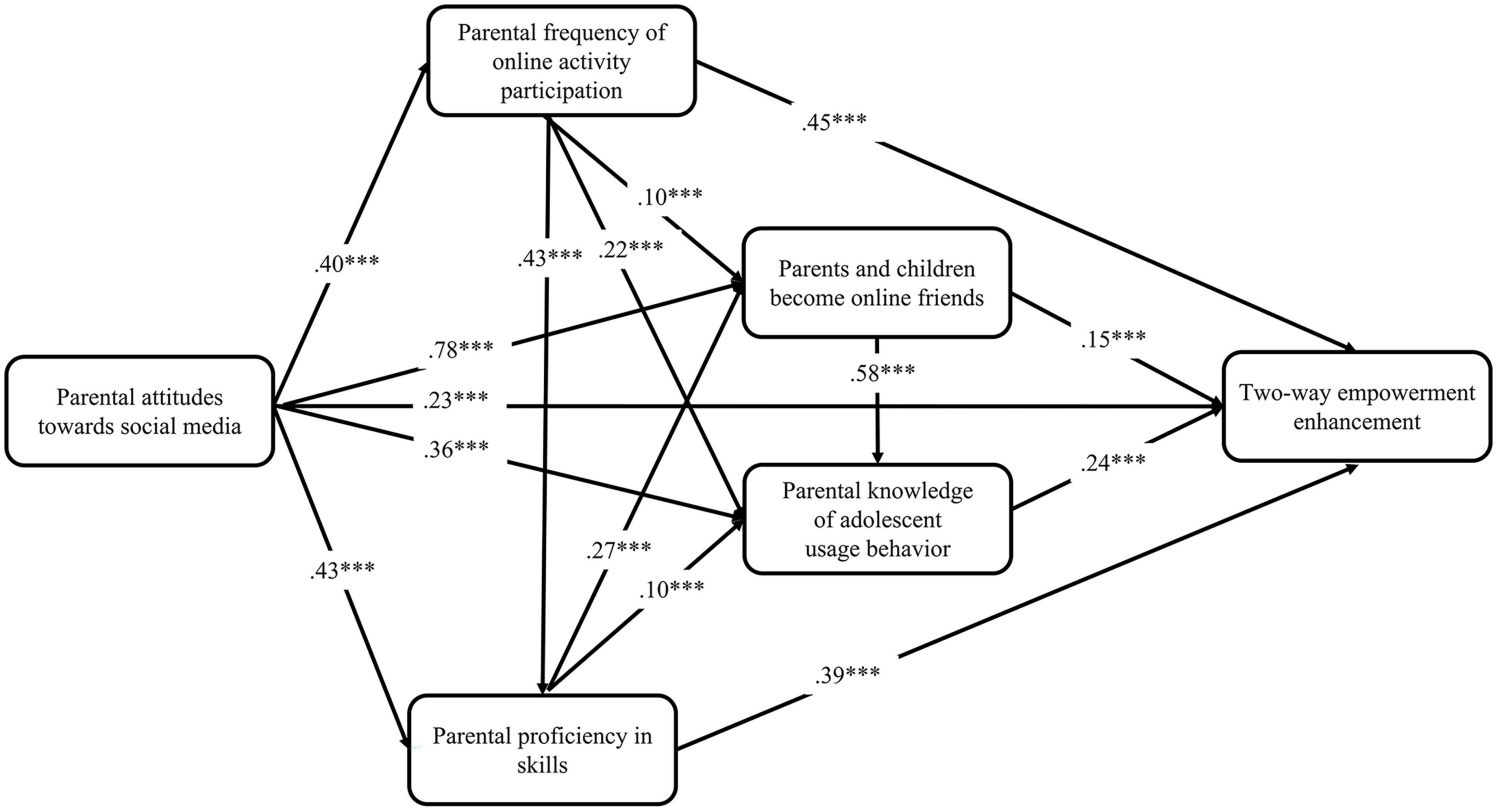
Predictive path coefficients of two-way empowerment enhancement. *p < 0.05, **p < 0.01, ***p < 0.001.

The structural equation modeling from an empirical perspective reveals the dynamics of the adoption of different parental interventions, and presents the relationships among different possible factors. The aforementioned study proposed four dimensions of parental intervention based on which supplementary research hypotheses were proposed and tested. The fixed-load and maximum likelihood methods were adopted to estimate the previously constructed theoretical model of parental intervention; the standardized path coefficients are shown in Figures 2–5.

The results show that all the fit indices of the four prediction models were extremely good (Table 1), and all the indicators of the models satisfied the fit requirements of the structural equation model, indicating that the hypothetical model could be accepted.

**Table 1:** Fitting results of the prediction model of parental-mediated intervention strategies.

Model	X^2^/df	RMSEA	PNFI	PGFI	GFI	AGFI	CFI	NFI	IFI	TLI
Rule-based technical enhancement	2.56	0.07	0.79	0.88	0.97	0.90	0.97	0.97	0.97	0.94
Active guidance enhancement	2.31	0.05	0.76	0.84	0.98	0.90	0.98	0.98	0.98	0.92
Informed participation enhancement	2.78	0.07	0.74	0.82	0.96	0.91	0.97	0.96	0.96	0.91
Two-way empowerment enhancement	2.06	0.07	0.73	0.83	0.98	0.92	0.98	0.98	0.98	0.90

From the perspective of explanatory power, parents’ attitudes toward social media, frequency of online activity participation, proficiency in skills, and other factors jointly play a role in the dependent variables. Parents’ attitude toward social media not only had a direct effect on the dependent variable, but also indirectly influenced the dependent variable through the mediating variables “frequency of online activity participation,” “proficiency in skills,” “parents’ knowledge of their children’s use behavior,” and “becoming online friends.” The estimation results from the prediction models show that our previous predictions—15 research hypotheses—were supported to varying degrees by the empirical data in all four models. And digital family environment constitutes the intermediary chain between individual technical characteristics and parental intervention. A comparison of the four models shows that the explanatory power of the individual’s technical characteristics and digital home environment for the dependent variables together differed more significantly between the rule-based technical enhancement (R^2^ = 0.35), active guidance enhancement (R^2^ = 0.38), informed participation enhancement (R^2^ = 0.56) and two-way empowerment enhancement models (R^2^ = 0.83).

Comparatively, the two-way empowerment enhancement model fits better with the current reality of parental intervention on children’s media use in digital families. It has higher explanatory power, which can be regarded as an ideal family digital literacy enhancement strategy. Since the harmony of the online family environment is a prerequisite for the parental knowledge of the offspring’s use behavior, the introduction of the online family environment variable is more explanatory for the informed participation model. Rule-based technical enhancement and active guidance enhancement, which were born out of restrictive and active interventions in the traditional media era, are far less effective than the two aforementioned models in explaining digital literacy enhancement.

## Discussion

### Conclusion and implications

The Covid-19 pandemic has induced adolescents’ increased engagement on social media for multiple activities: work (Liu et al., 2022e), education (Hsu et al., 2021), socialization (Liu et al., 2021), entertainment (Chen et al., 2022a,b), etc., which may influence parental intervention strategies. Our study focused on parental intervention in the post-pandemic era. First, the concept of literacy improvement based on interaction extends beyond the interpretation of parental intervention and provides a comprehensive conceptual model with verifiable measurement dimensions. Second, the comparison based on the parent–child sample reconfirmed that the frequent occurrence of the four intervention strategies in the family and parent–child subjects has a certain degree of consensus. Third, the prediction model of “individual technical characteristics-online family environment-parent-mediated intervention” was constructed and verified, and the influencing factors were systematized. In contrast to previous explorations of hardware technology adoption and parent–child relationship quality as causal factors influencing parental intervention environments, an online family environment (“whether they are friends online” and “parents’ knowledge of their children’s use”) was introduced based on a parent–child interaction perspective to fit the new scenario of social media use.

### Theoretical implications

The theoretical implications of this study include moving beyond “protectionism” to “interactive literacy enhancement,” innovating the concept and practice of parental intervention in the post-epidemic era. Parental interventions in social media use should be to “guide” rather than “prohibit.” Parents should focus on “safe use” instead of “better use,” effectively integrate “parent–child online interaction,” and implement interactive and guided parental interventions with a more inclusive and open mind. For example, parents should spend more time to find good “digital projects” for the family and share their digital technology skills with their children to promote the digital literacy of both parents and children and build a new communication pattern for a digital family.

### Practical implications

Summing up the hierarchical progressive logic of parental intervention, family cohesion is the “anchor point” of media intervention. This study not only provides an empirical description of parental interventions, but also reveals that parents’ technological characteristics and online family environment jointly influence the occurrence of parental interventions. The online family environment constitutes a mediating chain between individual technological characteristics and the occurrence of parental interventions. Parents’ technological characteristics are also antecedent variables in the construction of the online family environment. In summary, parents guide their children’s social media use with the concept of literacy improvement, and they are not only observers but also active participants in their children’s online activities. The effectiveness of the intervention is more because of the “cohesion” between parent–child subjects.

### Limitations and directions for future

From a theoretical perspective, future research should focus on the following: First, parents’ intervention in their children’s social media use varies, depending on the type of media; our study focused on a broad category of social media and did not distinguish between specific types. Subsequent refinement of the study is required. Second, although adolescents are considered digital natives, a few individuals may lack the ability to use social media effectively; this intra-generational disparity can be partly attributed to the lack of effective parental intervention. Thus, it is necessary to focus on the relationship between parental intervention and intra-generational inequality among adolescents. Third, our study confirmed the influence of individuals’ technological characteristics and online family environment on parental interventions; however, it did not involve the social and emotional decision-making process when parents adopted specific intervention methods. Subsequent attempts could be made to incorporate integrative behavioral models into the study of parental interventions.

## Data availability statement

The data analyzed in this study is subject to the following restrictions: some adolescent respondents asked for personal privacy protection. Requests to access these datasets should be directed to the corresponding author JC, chenjiali0907@163.com.

## Ethics statement

Written informed consent was obtained from the individual(s), and minor(s)’ legal guardian/next of kin, for the publication of any potentially identifiable images or data included in this article.

## Author contributions

BW: research design, conceptualization, data collection and analysis, manuscript writing, and supervising. JC: theory construction, data collection and analysis, manuscript writing, and supervising. All authors contributed to the article and approved the submitted version.

## Funding

This work was supported by a major project of the Education Science Fund of Jiangxi Province “Research on the improvement of minors’ digital literacy based on the use of social media” (20ZD010), and by Nanchang University Research Initiation Fund (9140/06301576).

## Conflict of interest

The authors declare that the research was conducted in the absence of any commercial or financial relationships that could be construed as a potential conflict of interest.

## Publisher’s note

All claims expressed in this article are solely those of the authors and do not necessarily represent those of their affiliated organizations, or those of the publisher, the editors and the reviewers. Any product that may be evaluated in this article, or claim that may be made by its manufacturer, is not guaranteed or endorsed by the publisher.
